# Transdiagnostic connectome signatures from resting-state fMRI predict individual-level intellectual capacity

**DOI:** 10.1038/s41398-022-02134-2

**Published:** 2022-09-06

**Authors:** Xiaoyu Tong, Hua Xie, Nancy Carlisle, Gregory A. Fonzo, Desmond J. Oathes, Jing Jiang, Yu Zhang

**Affiliations:** 1 Department of Bioengineering, Lehigh University, Bethlehem, PA USA; 2 Department of Psychology, University of Maryland, College Park, MD USA; 3 Department of Psychology, Lehigh University, Bethlehem, PA USA; 4 Center for Psychedelic Research and Therapy, Department of Psychiatry and Behavioral Sciences, Dell Medical School, The University of Texas at Austin, Austin, TX USA; 5 Center for Neuromodulation in Depression and Stress, Department of Psychiatry, University of Pennsylvania Perelman School of Medicine, Philadelphia, PA USA; 6 Departments of Pediatrics and Psychiatry, Carver College of Medicine, University of Iowa, Iowa, IA USA

**Keywords:** Predictive markers, Psychiatric disorders

## Abstract

Medication and other therapies for psychiatric disorders show unsatisfying efficacy, in part due to the significant clinical/ biological heterogeneity within each disorder and our over-reliance on categorical clinical diagnoses. Alternatively, dimensional transdiagnostic studies have provided a promising pathway toward realizing personalized medicine and improved treatment outcomes. One factor that may influence response to psychiatric treatments is cognitive function, which is reflected in one’s intellectual capacity. Intellectual capacity is also reflected in the organization and structure of intrinsic brain networks. Using a large transdiagnostic cohort (*n* = 1721), we sought to discover neuroimaging biomarkers by developing a resting-state functional connectome-based prediction model for a key intellectual capacity measure, Full-Scale Intelligence Quotient (FSIQ), across the diagnostic spectrum. Our cross-validated model yielded an excellent prediction accuracy (r = 0.5573, *p* < 0.001). The robustness and generalizability of our model was further validated on three independent cohorts (*n* = 2641). We identified key transdiagnostic connectome signatures underlying FSIQ capacity involving the dorsal-attention, frontoparietal and default-mode networks. Meanwhile, diagnosis groups showed disorder-specific biomarker patterns. Our findings advance the neurobiological understanding of cognitive functioning across traditional diagnostic categories and provide a new avenue for neuropathological classification of psychiatric disorders.

## Introduction

Current clinical neuroscience research generally relies on consensus-based diagnostic criteria such as DSM-5 [1] and ICD-10 [2]. These diagnostic criteria are mainly based on patients’ self-reports and clinician assessment of behavior, instead of neuropathological abnormalities, in part due to the elusive mechanisms of psychiatric disorders [3, 4]. In the past decade, a growing number of studies have suggested that consensus-based diagnosis criteria fail to address the heterogeneity and high comorbidity rates in psychiatric disorders [5–7], leading to a limited understanding of psychopathology which likely contributes to the suboptimal clinical efficacy of therapies [8]. To break the shackles tied by the case-control diagnosis framework, numerous recent studies sought to construct new dimensions of psychiatric disorders as advocated by the NIMH RDoC framework [9]. Using machine learning techniques, approaches have included defining disorder subtypes based on neuroimaging biomarkers identified by supervised dimensionality reduction [10–13] or unsupervised clustering [13–18] and data-driven neuroimaging analysis across diagnosis boundaries [19]. Furthermore, recent studies pioneered techniques to extract individual components of each subject from neuroimaging-based connectome [20–22]. As result, robust correlations have been found between brain connections and cognitive measures in a broad range of diagnoses [23], demonstrating the possibility of making the individualized prediction of cognitive measures with neuroimaging data in the transdiagnostic population.

However, the reproducibility and reliability of these emerging results are limited by the relatively small sample size of neuroimaging datasets [24], yielding only preliminary clinically-applicable prediction models. Moreover, most available neuroimaging studies follow a case-control design, of which results may not generalize to transdiagnostic populations. Non-transdiagnostic categorical studies essentially still rely on clinical consensus-based categorization, thus not significantly contributing to the neuropathological-based definition of psychiatric disorders. To address these challenges, recent data collection initiatives aim to collect transdiagnostic neuroimaging data in large sample sizes, including the Healthy Brain Network (HBN) Biobank [25] and Philadelphia Neurodevelopmental Cohort [26]. With these open-access datasets, we have an unprecedented opportunity to identify robust neuromarkers using functional neuroimaging data and develop reliable prediction models for cognitive assessment, diagnosis, prognosis, and treatment outcome [24].

To bridge the gap between machine-learning-based biomarker findings and consensus-based clinical practice, we developed a resting-state functional MRI (rsfMRI) connectome-based machine learning model to predict intellectual capacity in a transdiagnostic population at the individual level. Intellectual capacity, as quantified by intelligence quotient, is a common and widely-utilized measure that assesses both cognitive functions and acquired abilities and is highly predictive of important life outcomes such as educational achievement, job performance, and overall well-being [27, 28]. Intelligence is also reflected in the organization and function of brain networks [29]. Thus, it provides a useful summary of cognitive functioning with real-life predictive validity and biological relevance. Our connectome-based machine learning model was trained and cross-validated using large-scale data from the HBN Biobank [25] with 1721 subjects. The model yields a promisingly robust prediction performance which successfully generalized to three independent cohorts [30–32] with a total 2641 subjects (Table 1). We further identified interpretable connectome signature patterns that predicted the cognitive measure, which remarkably aligned with neurobiology findings for both the transdiagnostic population and each diagnosis group. Together, these efforts aim to identify neuroimaging-based cognitive biomarkers in transdiagnostic populations and propel the construction of new neuropathological-based definitions of psychiatric disorders, thus realizing personalized medicine and improving treatment outcomes.

**Table 1:** Discovery and replication cohorts.

		Discovery	Replication			Total
		HBN	ADHD-200	ABIDE I	ABIDE II	
*n*		1721	719	1030	892	4362
Gender	Male	1109	449	877	691	3126
	Female	612	269	153	201	1235
	Unknown	—	1	—	—	1
Diagnosis	HC	144	445	533	480	1602
	ADHD	811	274	—	—	1085
	ASD	90	—	497	412	999
	MDD	39	—	—	—	39
	Anxiety	189	—	—	—	189
	Other	438	—	—	—	438
Age	Mean	10.28 ± 2.61	11.94 ± 2.88	16.75 ± 7.78	15.67 ± 9.42	13.18 ± 5.26
FSIQ/FIQ	Mean	99.13 ± 15.94	110.79 ± 13.88	108.45 ± 14.93	111.16 ± 15.36	105.71 ± 15.23

## Methods

### Participants

This study used data from four independent cohorts, including Healthy Brain Network (HBN) [25], ADHD-200 [30], Autism Brain Imaging Data Exchange (ABIDE) I [31] and ABIDE II [32]. The HBN initiative, approved by the Chesapeake Institutional Review Board, recruited children and adolescents in age 5–21 at four study sites in the New York City area. Participants must have adequate verbal communication ability with the help of their parents or guardians. Subjects with severe neurological disorders, including severe impairment in cognitive (IQ < 66), acute encephalopathy, known neurodegenerative disorder, or other abnormalities that may prevent full participation in the protocol were excluded from recruitment [25]. The ADHD-200 dataset recruited children and adolescents with ADHD (*n* = 285) and health controls (*n* = 491) at eight study sites in age 7–21 [30]. The ABIDE I and II dataset included ASD patients and healthy people in age 7–64 at sixteen study sites. Inclusion and exclusion criteria for each of the study sites are available at https://fcon_1000.projects.nitrc.org/ [31, 32]. Informed consent was obtained from all subjects.

### Functional MRI data

The HBN protocol employed different fMRI scanners for each of data collection phases (test phase, deployment phase I and deployment phase II) [25]. Test phase utilized 1.5 T Siemens Avanto scanner with 45 mT/m gradients in a mobile trailer, which was upgraded with 32 RF receive channels, the Siemens 32-channel head coil and the University of Minnesota Center for Magnetic Resonance Research (CMRR) simultaneous multi-slice echo planar imaging sequence [25, 33]. Deployment phase I utilized 3.0 T Siemens Tim Trio scanner with a Siemens 32-channel head coil and the CMRR simultaneous multi-slice echo planar imaging sequence. Deployment phase II utilized 3 T Siemens Prisma scanner and the imaging sequence protocols was harmonized to the NIH ABCD Study. rsfMRI scans were recorded with duration greater than 10 minutes [25]. rsfMRI data in the ADHD-200 dataset were collected with varying protocols and scanner parameters specific to each of study sites. Notably, participants were asked to obey different constraints in study sites. For instances, during the rsfMRI data collection, participants at Oregon Health & Science University were instructed to stay still while keeping their eyes open and fixating on a standard fixation cross in the center of the display, whereas participants at Peking University were asked to relax and stay still, while either keeping their eyes open or closed in front of a black screen with white fixation cross displayed during the scan (https://fcon_1000.projects.nitrc.org/). rsfMRI data in ABIDE datasets are contributed by ADHD-200 Consortium members which conduct autism research (17 international sites for ABIDE I, 19 international sites for ABIDE II) and investigators willing to openly share rsfMRI data from individuals with ASD. Detailed rsfMRI scanning protocols and scanner parameters for each of the study sites are available at https://fcon_1000.projects.nitrc.org/ [31, 32].

### Phenotypical data

HBN, ADHD-200 and ABIDE possess a collection of psychiatric, behavioral, cognitive, and demographical phenotypes [25, 30–32]. For the main study, FSIQ assessed by Wechsler Intelligence Scale for Children Fifth Edition (WISC-V) [34] from the HBN dataset was utilized as the predictive cognitive measure. We also retrieved FSIQ subdomains (WMI, FRI, VCI, VSI, PSI) for interpretable analysis. For generalizability analysis on ADHD-200 and ABIDE, we used Full IQ as the equivalent to WISC-V FSIQ. Full IQ was assessed in different measures including Wechsler Intelligence Scale for Children other editions (WISC-II [35], WISC-III [36], WISC-IV [37]), Wechsler Abbreviated Scale of Intelligence (WASI) [38], Wechsler Intelligence Scale for Chinese Children-Revised (WISCC-R) [39], Differential Ability Scales II - School Age (DAS II) [40], Wechsler Adult Intelligence Scales (WAIS) [41], Hamburg-Wechsler Intelligence Test for Children (HAWIK-IV) [42] and Groninger Intelligence Test (GIT) [43]. Demographics (age, gender) from each dataset were also retrieved to investigate potential confounders. Subjects with missing values in these selected phenotypical variables were excluded from the study.

### Functional MRI pre-processing

The fMRI preprocessing was performed using fMRIPrep [44]. The T1-weighted image was corrected for intensity non-uniformity and then skull-stripped. Brain tissue segmentation of cerebrospinal fluid, white matter and gray matter was performed on the brain-extracted T1-weighted image using FSL [45, 46]. Volume-based spatial normalization was performed through nonlinear registration using brain-extracted versions of both the T1-weighted image and template. For each fMRI scan, the following preprocessing was performed. First, a reference volume and its skull-stripped version were generated using a custom methodology of fMRIPrep. A deformation field to correct for susceptibility distortions was estimated based on fMRIPrep’s fieldmap-less approach. Registration is performed with antsRegistration, and the process regularized by constraining deformation to be non-zero only along the phase-encoding direction, and modulated with an average fieldmap template. Based on the estimated susceptibility distortion, a corrected echo-planar imaging reference was calculated for a more accurate co-registration with the anatomical reference. The blood-oxygenation-level-dependent (BOLD) reference was then co-registered to the T1-weighted image. Co-registration was configured with 12 degrees of freedom to account for distortions remaining in the BOLD reference. Head-motion parameters with respect to the BOLD reference are estimated before any spatiotemporal filtering. BOLD signals were slice-time corrected and resampled onto their original space by applying a single, composite transform to correct for head-motion and susceptibility distortions. The BOLD signals were then spatially normalized into the standard space. Automatic removal of motion artefacts using independent component analysis was performed on the pre-processed BOLD on the Montreal Neurological Institute space time series after removal of non-steady state volumes and spatial smoothing with an isotropic, Gaussian kernel of 6 mm full-width half-maximum. Regional pairwise fMRI connectivity was calculated with the preprocessed fMRI time series based on 100 ROIs defined by the Schaefer atlas [47]. Subject information with qualified pre-processed rsfMRI data for each of the datasets was summarized in Table 1.

### Connectome-based predictive modeling (CPM)

CPM is a recently developed method for the identification of functional brain connections that significantly correlates to the behavior variable of interest [48], thus reducing the feature dimensionality [49–52]. First, we correlated FSIQ (or FIQ in generalizability analysis) with each of the connections across subjects using Pearson’s correlation within each cross-validation fold. Only the connections that are significantly correlated with FSIQ were retained for the predictive modeling analysis. A threshold of P-value was then applied to determine which edges were correlated to IQ. The P-value threshold of 0.05 was selected for further analysis to construct a prediction model with decent performance and reasonable computation cost. To further refine the feature set and build a robust prediction model, we adopted LASSO regression [53, 54], a well-known machine learning technique with sparsity constraint.

For the FSIQ prediction model training, CPM-selected IQ-correlated functional brain connections were fed into the LASSO model. Ten rounds of five fold cross-validation were conducted to evaluate the model performance and a unified model was constructed by averaging feature weights of each selected connection across the total 50 cross-validation models. An inner-loop cross-validation was further performed to find an appropriate hyperparameter for the LASSO model. For subjects who had two runs of fMRI scans, their two rsfMRI runs were either both in the straining set, or both in the cross-validation set. For each subject in the test set, the prediction performance was averaged on all available runs of fMRI scans from the subject. Permutation tests were further performed to confirm the statistical significance of the identified connectome signatures for FSIQ prediction. The permutation test was conducted by randomly permuting FSIQ values of subjects in training set and subsequently training prediction models with permuted FSIQ labels while this entire procedure was repeated for 1000 times. We acquired a P-value smaller than 0.001 for the permutation test since the predictive model had higher predictability than all 1000 permutation models (inset in Fig. 2a).

### ROI and network importance

The importance of an ROI with respect to FSIQ was defined as the average of feature weights of all functional brain connections involving the ROI. The importance of a brain network for FSIQ prediction was defined as the average of feature weights of all functional brain connections involving the network, including both within- and between-network connections, where the feature coefficients were retrieved from the unified FSIQ prediction model.

### Relationship between connectome signatures and IQ subdomains

To evaluate the correlation between functional connections and IQ subdomains, multiple linear regression (MLR) models predicting each of IQ subdomains were fitted with IQ-correlated functional connections identified by the unified FSIQ prediction model. For each MLR model, only 500 functional connections were incorporated which were either all the IQ-correlated connections or 500 randomly selected IQ-uncorrelated connections. For each of the IQ subdomains, the correlation with functional connectome was assessed by computing Pearson’s correlation coefficients between actual and predicted IQ subdomain measure.To evaluate the correlation between ROIs and IQ subdomains, MLR models predicting each of IQ subdomains were fitted with ROI importances as regressors. ROI importances were acquired from feature weights of functional connections in the unified FSIQ prediction model. ROI importances were calculated either using only IQ-correlated functional connections or all connections, respectively yielding IQ-correlated ROI importances and full-connectome ROI importances with full connectome. The correlations between full connectome ROI importances and IQ subdomains were employed as a reference to confirm the significance of correlation between IQ-correlated ROI importances and IQ subdomains. The correlations were eventually assessed by computing Pearson’s correlation coefficients between actual and predicted IQ subdomain indices from MLR models.

### Individual differentiability

The individual differentiability (*ID*) was employed to assess the variance in subject-level rsfMRI functional connectome. A mean connectome was first calculated as the mean of functional connectivity across subjects. Notably, the mean connectome was calculated based on all subjects instead of healthy subjects to ensure the identified variability was shared by the entire transdiagnostic population, aligning with the primary goal of this study. Thereafter, the individual differentiability of i th subject was defined as the sum of absolute differences of each j th functional connection between the subject’s connectome and the mean connectome:

1 $$ID_i = \mathop {\sum}\limits_{j = 1}^{n_{FC}} {\left| {FC_{ij} - \frac{{\mathop {\sum}\nolimits_{i = 1}^{n_{subjects}} {FC_{ij}} }}{{n_{subjects}}}} \right|}$$

where *FC*_ij_ is the feature coefficient of j th functional connection for i th subject. The individual differentiability hence reflects the deviation of each subject’s connectome to the mean connectome. Thus the distribution of individual differentiability in a population group of interest indicates the variance of functional connectome in that population group. Similar ideas of individual connectome fingerprint and differential identifiability were explored in recent studies [20–22, 55].

### Reproducibility on independent cohorts

An extensive replication analysis was conducted to assess the reproducibility of the identified brain connectome signatures on other independent cohorts. A unified FSIQ prediction model was developed on the HBN dataset by averaging feature weights of functional brain connections in each cross-validation fold. The unified FSIQ prediction model was then applied to the ADHD-200, ABIDE I and ABIDE II datasets, respectively. For each of the replication datasets, the prediction performance was assessed by computing Pearson’s correlation between the predicted FSIQ values and the real measures. To confirm the statistical significance of the model prediction on replication cohorts, we trained another 1000 permutation models on the HBN dataset. The permutation models were also developed using cross-validation to align with the procedure for predictive model training. Each of the permutation models was derived by randomly permuting FSIQ values of subjects in the training set of each cross-validation fold and then averaging all models for individual cross-validation folds. Afterwards, the 1000 unified permutation models were applied to the three independent cohorts, to evaluate the significance of predictive models. For all the three cohorts, the HBN-based prediction model performed significantly better (*p* < 0.001) than the random permutation results (Fig. 5).

## Results

### Transdiagnostic connectome signatures predictive for individual intellectual capacities

**Fig. 1:**
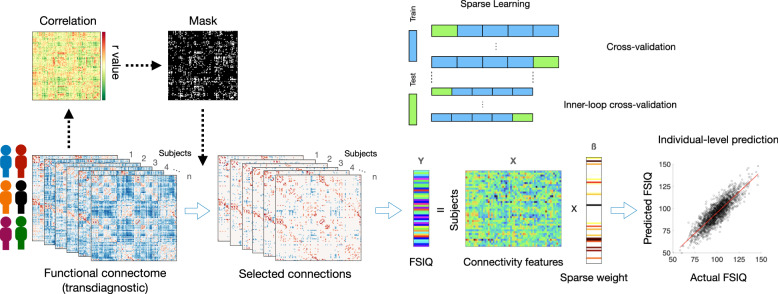
A flowchart of the transdiagnostic predictive modeling for intellectual capacity. First, the functional connectome is constructed using resting-state fMRI data of individuals from multiple diagnostic groups. Then, connectome-based predictive modeling (CPM) applies a mask on the functional connectome based on its correlation with intellectual capacity, quantified by Full-Scale Intelligence Quotient (FSIQ), to reduce feature dimensionality. A sparse learning algorithm (LASSO) with cross-validation further selects predictive connections and derives feature weights to generate individual-level prediction of FSIQ.

**Fig. 2:**
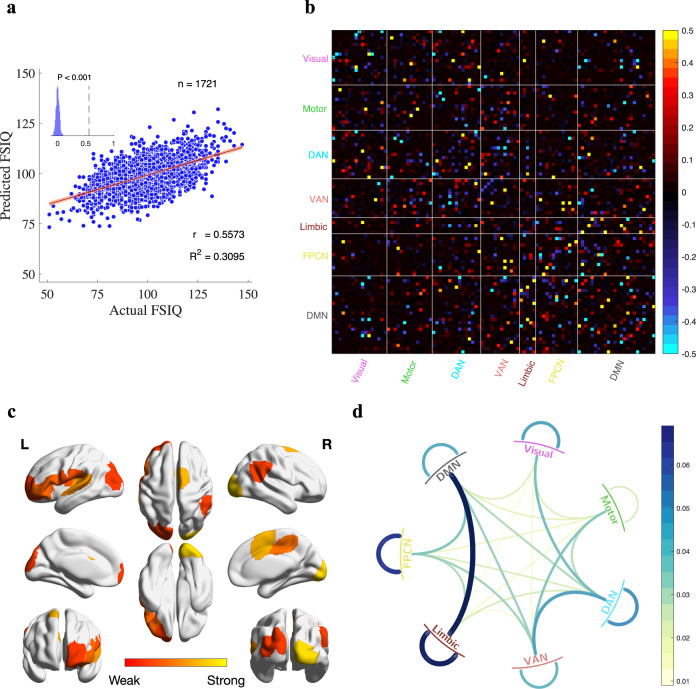
Transdiagnostic connectome signatures predictive for individual FSIQ. **a** FSIQ prediction model on transdiagnostic population. The model is evaluated by 10x five-fold cross-validation. The inset displays the null distribution of model performance by permutation testing with 1000 permutations. Dashed line indicates actual performance. Error bar shows standard deviation. This model is hereafter referred to as the “standard model’. **b** Edge-wise importance of functional brain connections with respect to FSIQ. Each cell represents the edge-wise importance derived by averaging feature weights of FSIQ prediction models for each of cross-validation folds. Red/blue cells indicate positive/negative feature weights of brain connection with respect to FSIQ. **c** ROI importance with respect to FSIQ. ROI importance is derived by averaging the absolute feature weights of all functional brain connections involving an ROI. The top 10 important ROIs are shown for clarity. **d** Importance of functional brain connections at the network level. The importance of functional brain connections between two networks/within a network is defined by the average of absolute feature weights of all functional brain connections residing between those two networks/within the network of interest.

We built prediction models using a brain connectome constructed with rsfMRI by combining CPM and LASSO to predict intellectual capacities on the large-scale transdiagnostic population (*n* = 1721) from the HBN Biobank [25] (Fig. 1). Full-Scale Intelligence Quotient (FSIQ) was selected as the quantitative measure of intellectual capacity and the model performance was evaluated by a 10x five-fold cross-validation (*r* = 0.5573, R-squared=0.3095, *p* < 0.001, Fig. 2a; permutation test-verified using 1000 permutations, *p* < 0.001). In subsequent analyses, this model is hereafter referred to as the “standard model”.

To further investigate which brain regions of interest (ROIs) and networks were responsible for FSIQ prediction, we examined the connectivity weights driven by the prediction model. The connections with large absolute weights were distributed over the entire brain (Fig. 2b). The unified model showed high FSIQ predictability (*r* = 0.8193, R-squared=0.6076, *p* < 0.001, Supplementary Fig. 1a), but it suffered from information leakage. Thus, we only assessed model performance using cross-validation results. Afterward, we empirically determined the top 500 strong connections as IQ-correlated connections to reduce dimensionality and facilitate interpretation. We confirmed that the prediction model with only IQ-correlated connections maintained reasonable FSIQ predictability on the entire transdiagnostic population (*r* = 0.7857, R-squared = 0.5539, *p* < 0.001, Supplementary Fig. 1b). Subsequently, the importance of each ROI was evaluated by averaging IQ-correlated connections involving the same ROI (Fig. 2d). We found that the visual cortex (striate and extrastriate cortices), part of the frontal lobe and supramarginal, superior parietal, angular gyri of the parietal lobe are the most contributive regions to FSIQ, aligning with the findings reported in a previous neuroscience study [56].

In addition, we interpreted the FSIQ connectome signature on the network level according to the Yeo’s seven networks [57] (Fig. 2e). The results showed high accordance with neuroscience findings [56, 58–63]. Specifically, connections within Limbic and between Limbic and default mode network (DMN) are the most influential ones in FSIQ prediction. The limbic-paralimbic-striatal network has been shown to regulate overall brain activation during tasks [60], and the posterior DMN is especially active during memory function [61], which in combination may be particularly relevant to broad cognitive capacities. The connections within the frontoparietal control network (FPCN) were also contributive for intelligence prediction, which echoes the parieto-frontal integration theory of intelligence [56, 58] and recent fMRI-based studies [62, 63]. In addition, the connections between/within visual and attention networks were associated with significant weights, demonstrating the importance of visual attention in measures of intelligence [58]. The connections between VAN and DMN with strong negative weights also were consistent with a prior relevant study [59]. These results together confirmed that the promising FSIQ prediction model we built was indeed achieved by the contribution of connections in neuroscience-recognized cognition-related brain regions.

Additionally, a leave-study-site-out analysis confirmed the generalizability of our prediction model across four study sites in the HBN dataset (number of subjects: *n*_1_ = 677, *n*_2_ = 53, *n*_3_ = 842, *n*_4_ = 149, Supplementary Fig. 2). The model derived without the left-out site showed comparably high performance as the model trained with data from all sites (Supplementary Figs. 3 and 4). We further verified that our prediction model was valid across gender and age (female subjects: *r* = 0.5639, R-squared=0.3143; male subjects: *r* = 0.5557, R-squared = 0.3066; age group I (age < 9): *r* = 0.4736, R-squared = 0.2090; age group II (age between 9 and 12): *r* = 0.5868, R-squared = 0.3373; age group III (age > 12): *r* = 0.6171, R-squared=0.3684. *p* < 0.001 for all subject groups. Supplementary Fig. 5). A detailed analysis of the relationship between model predictability and age can be found in Supplementary Fig. 6. Lastly, we discovered that training the model with additional fMRI scans enhanced the model predictability (prediction on single fMRI run: r=0.4847, R-squared=0.2143; prediction on two fMRI runs: *r* = 0.5732, R-squared = 0.3276; Fisher’s *z* = 3.37. *p* < 0.001 for both predictability and Fisher’s z test. Supplementary Fig. 7).

### Transdiagnostic and disorder-specific connectome patterns in intellectual capacity prediction

To further investigate the quantitative contribution of brain networks to FSIQ prediction and whether they contribute uniquely to subjects with different psychiatric disorders, we assessed the model predictability in each of the diagnosis groups. The diagnosis groups we examined include healthy control (HC), attention-deficit hyperactivity disorder (ADHD), autism spectrum disorder (ASD), major depressive disorder (MDD), and anxiety disorders. The rsfMRI connectome-based model was predictive of FSIQ values for all of these diagnosis groups (HC: *r* = 0.5277, R-squared = 0.1586; ADHD: *r* = 0.5268, R-squared = 0.2771; ASD: *r* = 0.6613, R-squared = 0.3919; MDD: *r* = 0.5194, R-squared = 0.2249; anxiety disorders: *r* = 0.5826, R-squared=0.2992. *p* < 0.001 for all diagnosis groups. Supplementary Fig. 8), suggesting that connectome signatures driven by the FSIQ prediction model were general to non-patients and across psychiatric disorders. The diagnosis-specific performance of the “standard model” was used as the baseline reference for subsequent analysis.

**Fig. 3:**
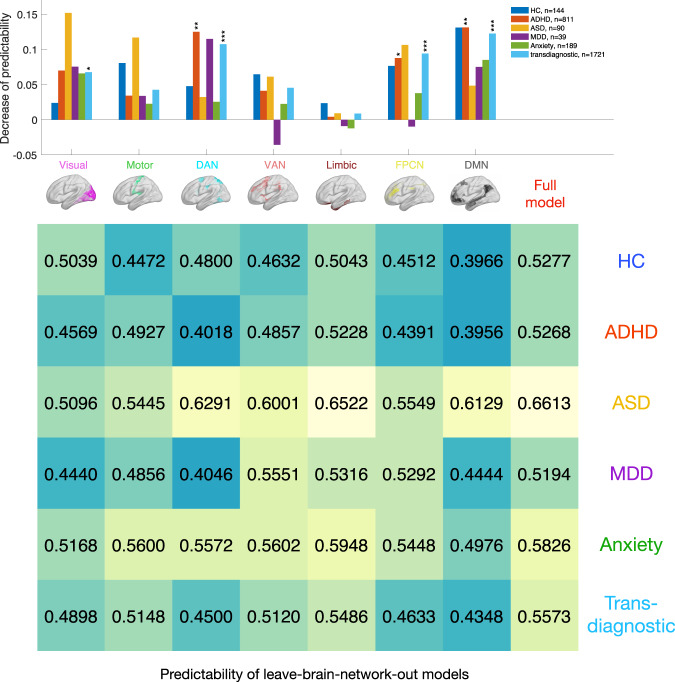
Transdiagnostic and diagnosis-specific connectome patterns in intellectual capacity prediction. The top bar plot displays the decrease of predictability for each diagnosis and brain network. The decrease of predictability is defined as the difference in prediction performance between the “standard model” and leave-brain-network-out model, where the feature weights involving the brain network of interest are set to zero. Here, rain networks include visual network, somatomotor network (Motor), dorsal-attention network (DAN), ventral-attention network (VAN), limbic network, frontoparietal control network (FPCN) and default-mode network (DMN). The diagnosis groups include healthy control (HC), attention-deficit hyperactivity disorder (ADHD), autism spectrum disorder (ASD), major depressive disorder (MDD), anxiety disorders and the whole transdiagnostic population. *** P_FDR_ < 0.001, ** P_FDR_ < 0.01, * P_FDR_ < 0.05. The brain maps indicate the corresponding brain region of each neural network. The bottom plot shows the predictability of leave-brain-network-out models and the “standard model” for each diagnosis group. Each of the brain networks is iteratively excluded from the prediction model by setting all feature weights involving the brain network of interest to zero. Boxed values indicate significant decrease after the removal of the corresponding brain network. For predictability, P_FDR_ < 0.001 for all diagnosis groups.

We further evaluated the importance of each brain network on predicting FSIQ for the entire transdiagnostic population as well as each diagnosis group by removing all functional connections involved in the brain network of interest from the prediction model. Resultant leave-one-network-out models were still predictive of FSIQ (Fig. 3, bottom table), which supported the idea that the broad concept of intelligence measured with a standardized instrument and a variety of subtests is distributively embedded across the brain thus enabling us to perform quantitative analysis on network importance. We defined the importance of a brain network as the decrease in the predictability of the network-removed prediction model compared with the model with the full connectome (Fig. 3). A set of Fisher’s z-tests comparing leave-brain-network-out models with the standard model identified brain networks with significant importance to the entire transdiagnostic population (Visual: Fisher’s z=2.73, P_FDR_=0.0110; Motor: Fisher’s z=1.75, P_FDR_=0.0935; DAN: Fisher’s z=4.23, P_FDR_=0.0004; VAN: Fisher’s z=1.86, P_FDR_=0.0881; Limbic: Fisher’s z=0.37, P_FDR_=0.7114; FPCN: Fisher’s z=3.73, P_FDR_=0.0005; DMN: Fisher’s z=4.78, P_FDR_=0.0004). The important networks identified by changes in predictability for the entire transdiagnostic population were well-aligned with the network strength acquired from feature weights of the prediction model (Fig. 2d): Visual network, DAN, FPCN, and DMN were the most influential networks, and removing any of them caused significant decrease in predictability. A more comprehensive analysis of diagnosis-specific connectome patterns can be found in Supplementary Table 2. Together, these observations demonstrate diagnosis-specific contributions of each brain network to intelligence, informing the association between identified transdiagnostic connectome signatures and the healthy-patient diagnostic spectrum.

### IQ-correlated biomarkers contribute to cognitive subdomains

**Fig. 4:**
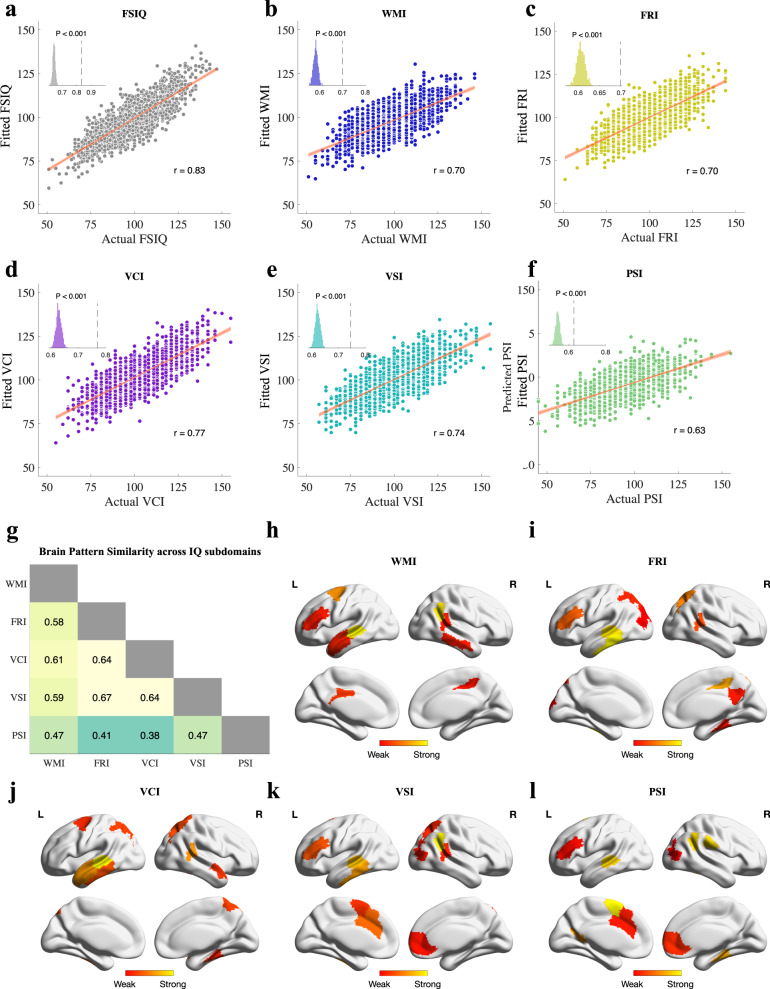
Correlations between IQ-correlated functional connections (n=500) and cognitive measures, including FSIQ and its subdomains. The correlations are calculated as the true-fitted correlation where the fitted values are derived by multiple linear regression models. **a** Full-Scale Intelligence Quotient. **b** Working Memory Index. **c** Fluid Reasoning Index. **d** Verbal Comprehension Index. **e** Visual Spatial Index. **f** Processing Speed Index. Each inset displays a distribution of correlation coefficients between cognitive measures and 500 randomly selected IQ-uncorrelated connections with 1000 trials. Dashed line indicates correlations between cognitive measure and IQ-correlated connections. Error bar shows standard deviation. **g** Brain pattern similarity across IQ subdomains. The similarity between each pair of subdomains is quantified as the correlation between feature weights derived by multiple linear regression models. Feature weights are normalized to ensure unit length. **h**–**l** ROI importance for each IQ subdomain. ROI importance is derived by summing the absolute feature weights of all functional brain connections involving an ROI. The top 10 important ROIs for each subdomain are shown for clarity.

To verify that the FSIQ prediction model indeed generated predictions based on interpretable links between rsfMRI connectome and intellectual capacity, we evaluated the correlation between the 500 IQ-correlated connections and FSIQ subdomains, including working memory index (WMI), fluid reasoning index (FRI), verbal comprehension index (VCI), visual spatial index (VSI) and processing speed index (PCI) [25]. Multiple linear regression showed significant correlations between IQ-correlated connections and FSIQ and its subdomains (FSIQ: *r* = 0.8334; WMI: *r* = 0.6983; FRI: *r* = 0.7521; VCI: *r* = 0.7703; VSI: *r* = 0.7439; PSI: *r* = 0.6268. *p* < 0.001 for FSIQ and all its subdomains. Figure 4a–f), suggesting the identified biomarkers were capable of predicting each aspect of intellectual capacity. Furthermore, a controlled permutation test was performed with 1000 trials by randomly and repeatedly selecting 500 connections from the 4450 IQ-uncorrelated connections. The controlled permutation test indicated that IQ-correlated connections possessed significantly higher correlations with FSIQ and its subdomains than IQ-uncorrelated connections (permutation test’s *p* < 0.001 for FSIQ and all of its subdomains, insets in Fig. 4), providing convincing evidence that the fMRI connectome-based information of IQ is indeed embedded and concentrated in the IQ-correlated connections identified by the prediction model. Additionally, to investigate whether IQ subdomains have common or unique neurobiological basis, we compared the similarity of brain patterns for each of IQ subdomains. The similarity between each pair of IQ subdomains was quantified as the correlation between feature weights derived from the multiple linear regression models (Fig. 4g). Interestingly, all pairs of subdomains showed some extent of similarity and difference (inner products between 0.3 and 0.7), suggesting these subdomains were indeed correlated yet supplementary aspects of intellectual capacity.

Next, we investigated whether this correlation between connectome and FSIQ/IQ subdomains was consistent at the level of ROI importance. The IQ-correlated ROI importance was calculated as the sum of weights of all IQ-correlated connections involving an ROI (Fig. 4h, l) and showed a significant correlation with FSIQ and IQ subdomains (FSIQ: *r* = 0.5492; WMI: *r* = 0.4455; FRI: *r* = 0.4803; VCI: *r* = 0.4936; VSI: *r* = 0.4692; PSI: *r* = 0.3464. *p* < 0.001 for all cognitive measures. Supplementary Fig. 9a–f). On the contrary, ROI importance calculated with the full connectome showed significantly lower correlation with IQ measures compared with IQ-correlated ROI importance (FSIQ: *r* = 0.3274, Fisher’s *z* = 8.13; WMI: *r* = 0.2814, Fisher’s z = 5.56; FRI: *r* = 0.2919, Fisher’s *z* = 6.53; VCI: *r* = 0.3182, Fisher’s z=6.19; VSI: r=0.2954, Fisher’s *z* = 6.00; PSI: *r* = 0.2601, Fisher’s z=2.79. p_correlation_ and p_Fisher_ < 0.001 for FSIQ and all its subdomains, except for p_Fisher_ < 0.01 for PSI. Supplementary Fig. 9g–l), indicating that it was a subset of functional connections, specifically the IQ-correlated connections identified by the prediction model, instead of a global tuning of ROIs, that predicted the intelligence of subjects. Meanwhile, the ROIs contributing to the correlation with FSIQ distributed across the whole-brain connectome. Together, these results suggested that IQ-predictive signatures were distributive at the ROI level but sparse at the connectivity level, echoing with the small-worldness theory in network neuroscience [64].

### Connectome signatures generalize to independent cohorts

**Fig. 5:**
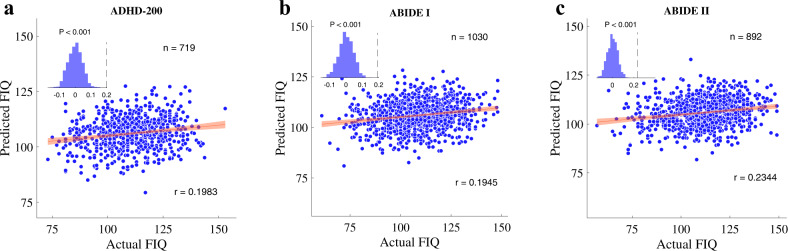
Reproducibility of FSIQ prediction model on independent cohorts. The cross-validated unified model trained on HBN was applied to three independent cohorts with different distributions of demographics, age and IQ. **a** The ADHD-200 dataset (*n* = 719). **b** The ABIDE I dataset (*n* = 1030). **c** The ABIDE II dataset (*n* = 892). Each inset displays the null distribution of model performance by permutation testing with 1000 permutations. Dashed line indicates actual performance. Error bar shows standard deviation.

Finally, we tested the generalizability of the developed prediction model to independent cohorts with different demographic, IQ, and diagnostic distributions, including ADHD-200 [30], ABIDE I [31] and ABIDE II [32]. The generalizability was verified by applying the FSIQ prediction model trained on HBN to the other three datasets. Encouragingly, the model derived on HBN showed significant predictability to IQ on all these three independent cohorts (ADHD-200: *r*=0.1983; ABIDE I: *r*=0.1945; ABIDE II: *r*=0.2344. *p* < 0.001 for all cohorts. Figure 5a–c). Additionally, random permutation tests of 1000 times were performed by applying permuted models trained on HBN to each cohort, further confirming that the predictability of our model was significant (p_permutation_<0.001 for all cohorts, insets in Fig. 5a–c) and generalizable to independent cohorts. To address potential concerns of site effect, we further compared the model performance yielded by unharmonized data and data harmonized with the ComBat technique [65, 66]. As a result, harmonized and unharmonized data showed very similar FSIQ predictability (Supplementary Table 3), demonstrating the HBN-trained model’s robust generalizability to independent cohorts.

## Discussion

In this study, we developed a rsfMRI connectome-based prediction model, with which we successfully revealed connectome signatures predictive for individual intellectual test scores in a large-scale transdiagnostic population. These identified biomarkers were capable of predicting intellectual capacities with rsfMRI data collected from a study site that was independent from the training set, which utilized a different fMRI acquisition configuration and with a possibly different FSIQ distribution. Moreover, these biomarkers were generalizable to independent cohorts from other studies with different scanning protocols, demographic distributions and diagnosis groups. This generalizability demonstrates the potential of our quantified connectome signatures to be applied in real-world clinical use for measuring individual cognitive function and subcategorization of psychiatric disorders with respect to cognitive dimensions. We also observed that training the prediction model with additional runs of rsfMRI scans significantly improved the prediction performance on individual subjects. This suggests that researchers or clinicians should collect multiple runs of fMRI scans sessions for each subject, if possible, to optimize the performance of quantitative analysis on cognition or other phenotypical behavior measures. More importantly, though recent studies have also reported the correlation between rsfMRI connectivity and cognitive behavior [67], or individual IQ prediction based on neuroimaging data [68–70], our present work, for the first time, developed a connectome-based FSIQ prediction model on a transdiagnostic population with high performance generalizable to independent cohorts, thus distinguished from previous studies and providing reliable results for the investigation into brain connectome-cognition relationship.

In addition, brain networks showed different contributions to intellectual capacities for each diagnosis group. We matched Yeo’s 7 networks [57] with Brodmann areas (BAs) to facilitate the direct comparison of our results with neuroscience studies. Remarkably, the diagnosis-specific network contribution we identified from this study accorded with results from previous non-transdiagnostic psychiatric studies. FPCN (BA9,46), Motor (BA6,7), DMN (BA8,24), and VAN (BA44) are brain networks with the most influential effects on IQ in healthy subjects [71]. Weaker connectivity in the prefrontal cortex (mainly consists of DAN, FPCN, and DMN) correlates to low IQ in children and adolescents with ADHD [72], and neurometabolic changes in the dorsolateral prefrontal cortex (mainly consists of DMN) correlate with IQ difference in patients of anxiety disorders [73]. Together, these results imply disorder-specific mechanisms of IQ measured abilities. Intriguingly, we noticed that the IQ-influential networks for each diagnostic category were highly overlapped with brain networks that distinguish HC from patients [74–77]. This observation inspires a new disorder subcategorization criterion: each psychiatric disorder can be classified into typical and atypical subtypes depending on whether a subject has neurobiological changes in the brain networks that show correlations with the disorder’s diagnosis, realized by assessing FSIQ since disorder-specific IQ changes somewhat reflect neurobiological changes in disorder-indicating networks. Typical and atypical patients possibly compose the heterogeneity of psychiatric disorders that we observed in clinical practice. Thus, distinguishing disorder subtypes in this way has the potential to guide intervention development for achieving personalized medicine and improved treatment outcomes. Furthermore, the effects of brain networks on IQ may also explain the homogeneity in different psychiatric disorders that patients diagnosed with different disorders may experience similar symptoms and respond to the same medications. Taken together, our results indicate the possibility and benefits of stratifying patients based on cognitive measures and treating them depending on neurobiological alterations instead of diagnosis labels.

Notably, though age was not correlated with IQ, it affected model predictability. We found that age groups with higher predictability also had lower normative-connectome-based individual differentiability. As we hypothesized that lower predictability may be due to higher variance in rsfMRI-based functional connectome, future studies can further quantitatively model the individual noise using individual differentiability. By incorporating individual differentiability as a prior constraint into connectome-based predictive modeling, we may obtain further improved prediction performance of cognitive behavior. Such a modified strategy may be applied to connectome-based predictive modeling of other variables, such as diagnosis classifiers and predictors of disorder-specific cognitive measures.

While numerous recent studies have demonstrated fMRI-based predictive models on the diagnosis of psychiatric disorders [78–83] and cognitive measures [24, 70, 84], the issue of large residuals of predicted values has not been completely solved, including in our present work. Future studies are required for better quantitative modeling to translate neuroimaging-based biomarker findings into clinical tools for diagnosis and treatment decisions. We consider the mean-connectome-based noise modeling a starting point to initiate these efforts. Future work is also required for confirming the diagnosis-specific FSIQ biomarker findings using a transdiagnostic population with more balanced subject numbers of each diagnosis group. An understanding of diagnosis-specific cognitive biomarkers can provide insight into how psychiatric disorders may develop from different brain network origins. Additionally, future studies can focus on the brain pattern of intellectual capacity subdomains, thus obtaining essential knowledge about detailed neurobiological basis of specific cognitive functions and potentially identify principle components in intelligence. Lastly but importantly, more advanced techniques to address site effect, such as transfer learning, may be employed to further improve the model performance on independent cohorts.

In summary, we developed a rsfMRI connectome-based FSIQ prediction model on a transdiagnostic population with large sample size and identified a signature pattern of rsfMRI functional connectome that was predictive of FSIQ. These results demonstrated the robust relationship between brain functional connectome and intellectual capacity across psychiatric disorders, which lit the way toward a novel dimensional disorder categorization based on neurobiological alterations and personalized treatment for psychiatric disorders.

## Supplementary Information

Supplementary Information

## Data Availability

Codes of the brain connectome analyses is available from the corresponding author upon request.
