# Supplementary material for: Transdiagnostic connectome signatures from resting-state fMRI predict individual-level intellectual capacity
Source: Transl Psychiatry. 2022 Sep 6;12:367. doi: 10.1038/s41398-022-02134-2 (PMC9448815; doi:10.1038/s41398-022-02134-2)
Supplement: Supplementary file 1 — Supplementary Information [file 41398_2022_2134_MOESM1_ESM.docx]

**Transdiagnostic Connectome Signatures from Resting-state fMRI Predict Individual-level Intellectual Capacity**

***Supplementary Information***

Supplementary Figure 1: Unified FSIQ prediction models

Supplementary Figure 2: Leave-study-site-out analysis of FSIQ prediction model

Supplementary Figure 3: Reference models for leave-study-site-out analysis

Supplementary Figure 4: Prediction performance difference across study sites.

Supplementary Figure 5: FSIQ prediction across gender and age

Supplementary Figure 6: Individual differentiability of functional connectome across age

Supplementary Figure 7: Prediction reliability enhanced by additional runs of rsfMRI scans Supplementary Figure 8: FSIQ prediction for each diagnosis group

Supplementary Figure 9: Correlations between ROI importance and cognitive measures

Supplementary Table 1: Diagnosis distribution across main study sites

Supplementary Table 2: Network importance with respect to FSIQ across diagnosis

Supplementary Table 3: Replication of FSIQ prediction on independent cohorts with data harmonization


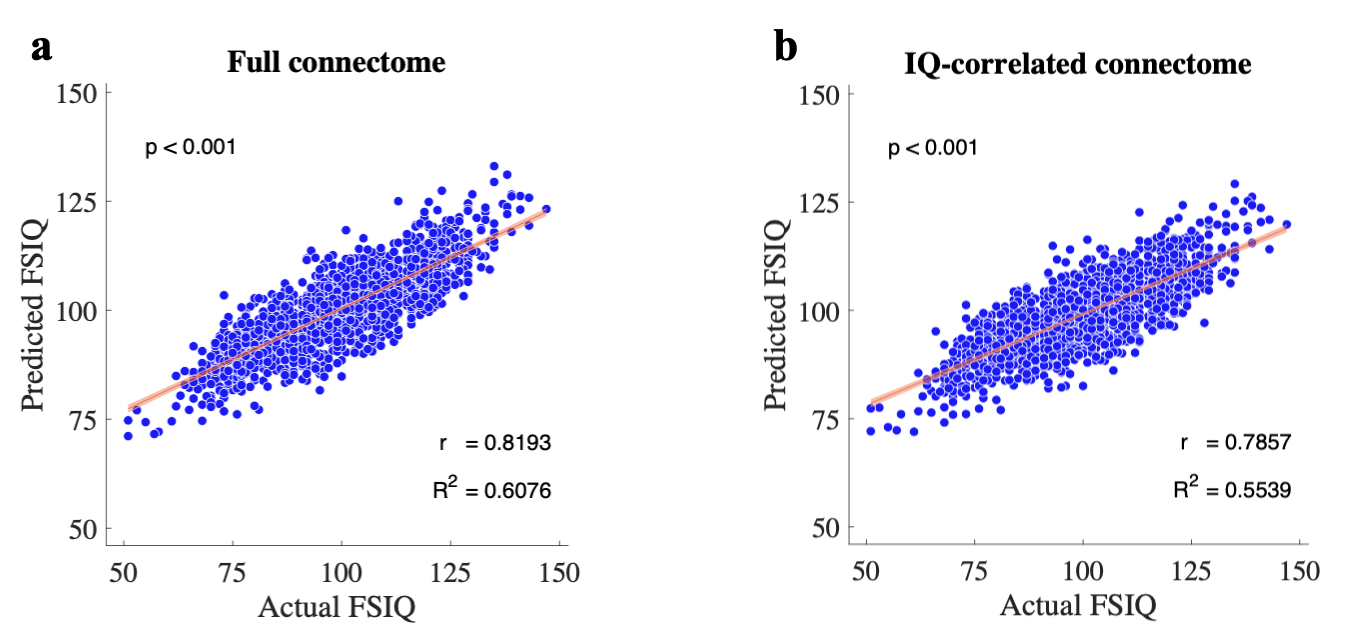


**Supplementary Figure 1 | Unified FSIQ prediction models.** Unified models are derived by averaging feature weights of functional brain connections in each of cross-validation folds. **(a)** All feature weights in the full connectome are incorporated. **(b)** Only feature weights of the top 500 FSIQ-correlated functional brain connections are included. Feature weights of FSIQ-uncorrelated functional brain connections are set to zero. Unified models suffer from the information leakage issue. They are only utilized to obtain correlations between FSIQ and full/a subset of brain connectome instead of evaluating model performance.


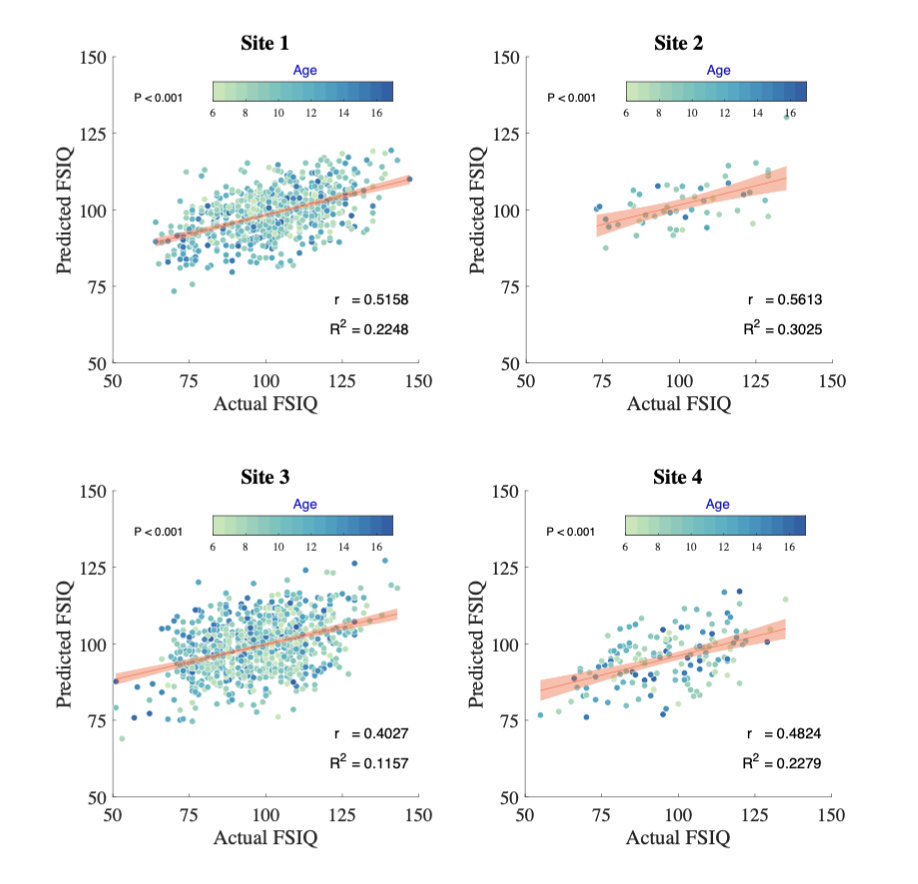


**Supplementary Figure 2 | Leave-study-site-out analysis of FSIQ prediction model.** Subjects from each of the study sites are iteratively and exclusively held out as test sets (Number of subjects: n_1_=677, n_2_=53, n_3_=842, n_4_=149). Subjects from the other three study sites are used as the training set to develop leave-study-site-out models. All leave-study-site-out models were significantly predictive of FSIQ values from the unseen study site. Ages of subjects are color-coded.


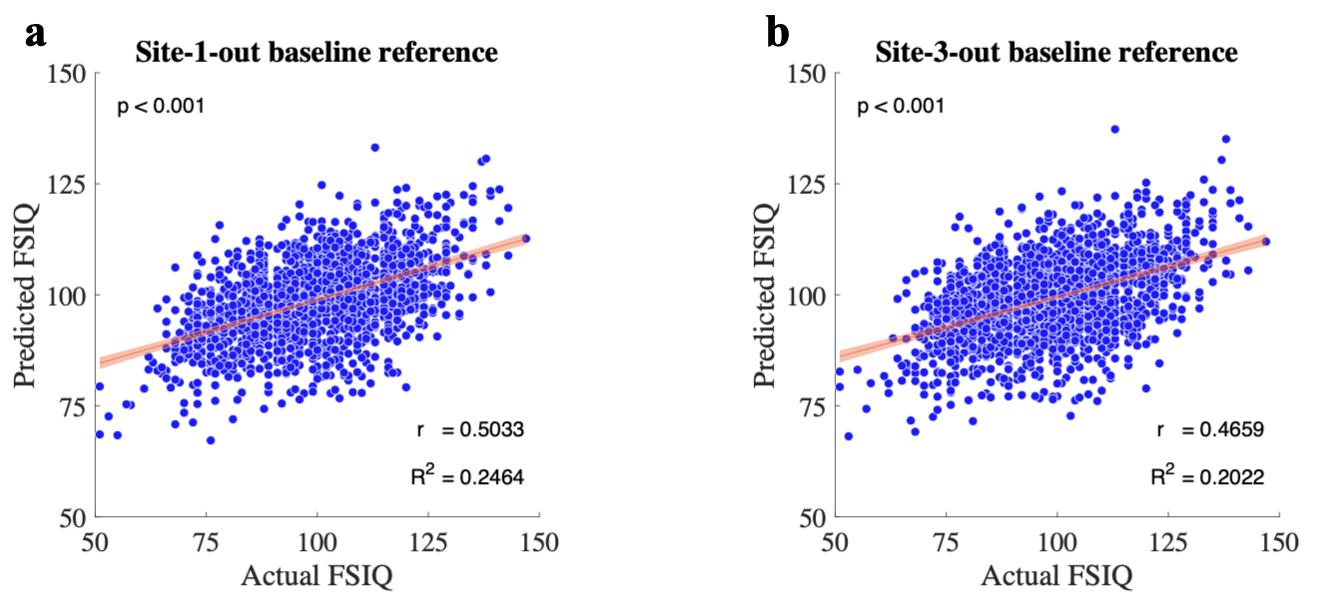


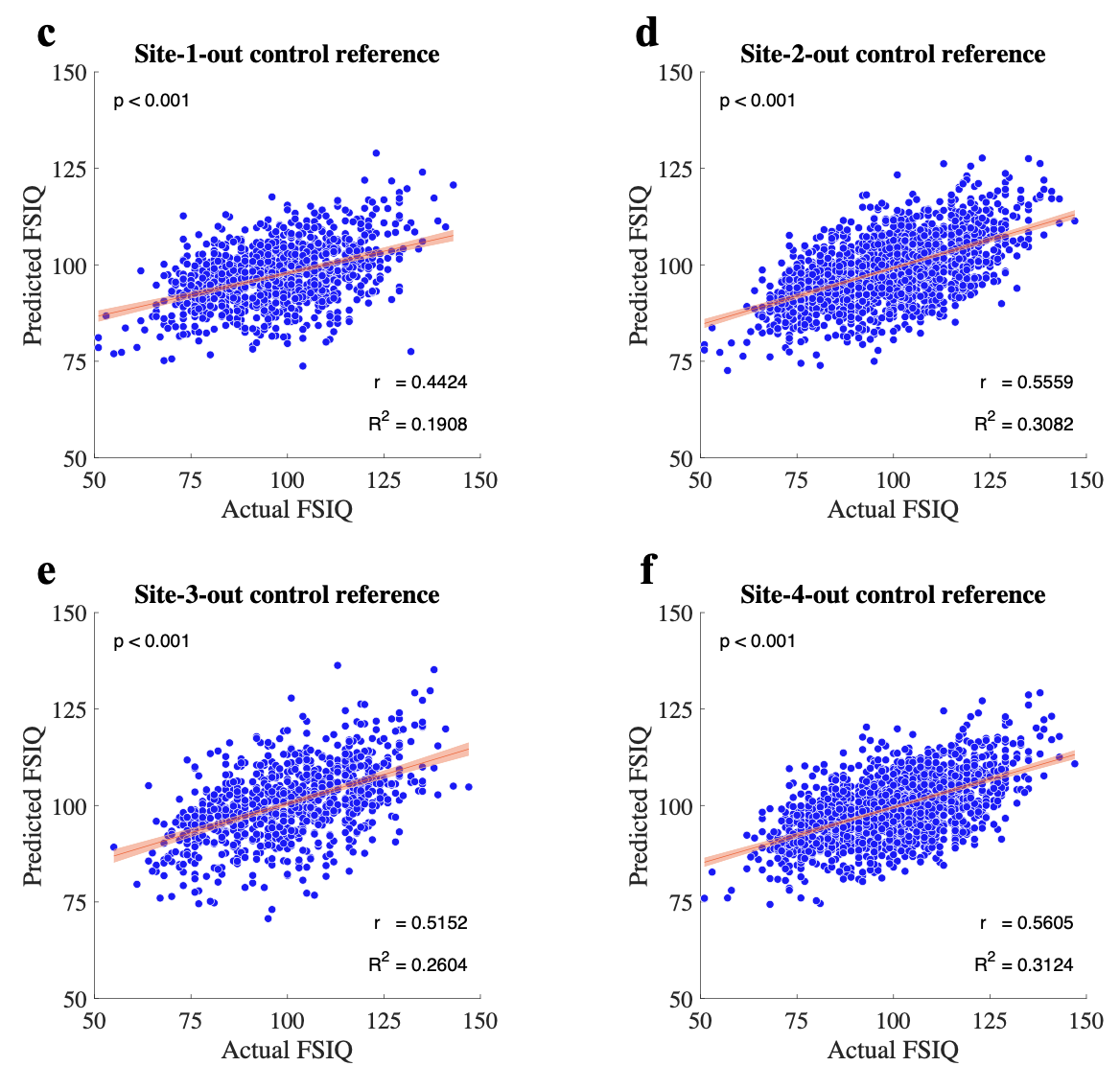


**Supplementary Figure 3 | Reference models for leave-study-site-out analysis.** To further evaluate the generalizability of our prediction model to data from unseen study sites, we used two sets of reference models. **(a-b)** First, to evaluate the predictability of leave-study-site-out models excluding the effect of training size decrease, a set of baseline reference models was derived by down-sampling the training set of the “standard model” to match the sizes of leave-study-site-out training set. For site 1 and site 3, they respectively consisted of 42% and 50% of the total samples, so we built additional corresponding training-downsampled models as their baseline references. The “standard model” was employed as the baseline reference for site 2 and site 4. Baseline reference models showed predictability similar to leave-study-site-out models (Fisher’s z test comparing leave-study-site-out and baseline reference models: Site 1: Fisher’s z=0.40, P=0.6892; Site 2: Fisher’s z=0.04, P=0.9681; Site 3: Fisher’s z=-1.85, P=0.0643; Site 4: Fisher’s z=-1.19, P=0.2340), demonstrating the model’s generalizability to data from unseen study site. **(c-f)** Second, we further evaluated the generalizability using a set of control references. Control reference used the same leave-study-site-out models as in Supplementary Figure 3, but applied the models on subjects from seen study sites. The results were then compared with predictions on subjects from the excluded study site to quantify the difference in model performance for same-sites data and unseen data. Encouragingly, the leave-study-site-out models generally showed similar performance on data from unseen study site compared with seen study sites (Fisher’s z test comparing leave-study-site-out model predictability on these two groups: Site 1: Fisher’s z=1.93, P=0.0536; Site 2: Fisher’s z=-0.39, P=0.6965; Site 3: Fisher’s z=-2.96, P<0.005; Site 4: Fisher’s z=-1.24, P=0.2150), suggesting that the FSIQ prediction model generally predicts unseen data as accurate as data from seen study sites. The only exception was site 3, for which the leave-study-site-out model predicts same-sites data better than unseen data.

**
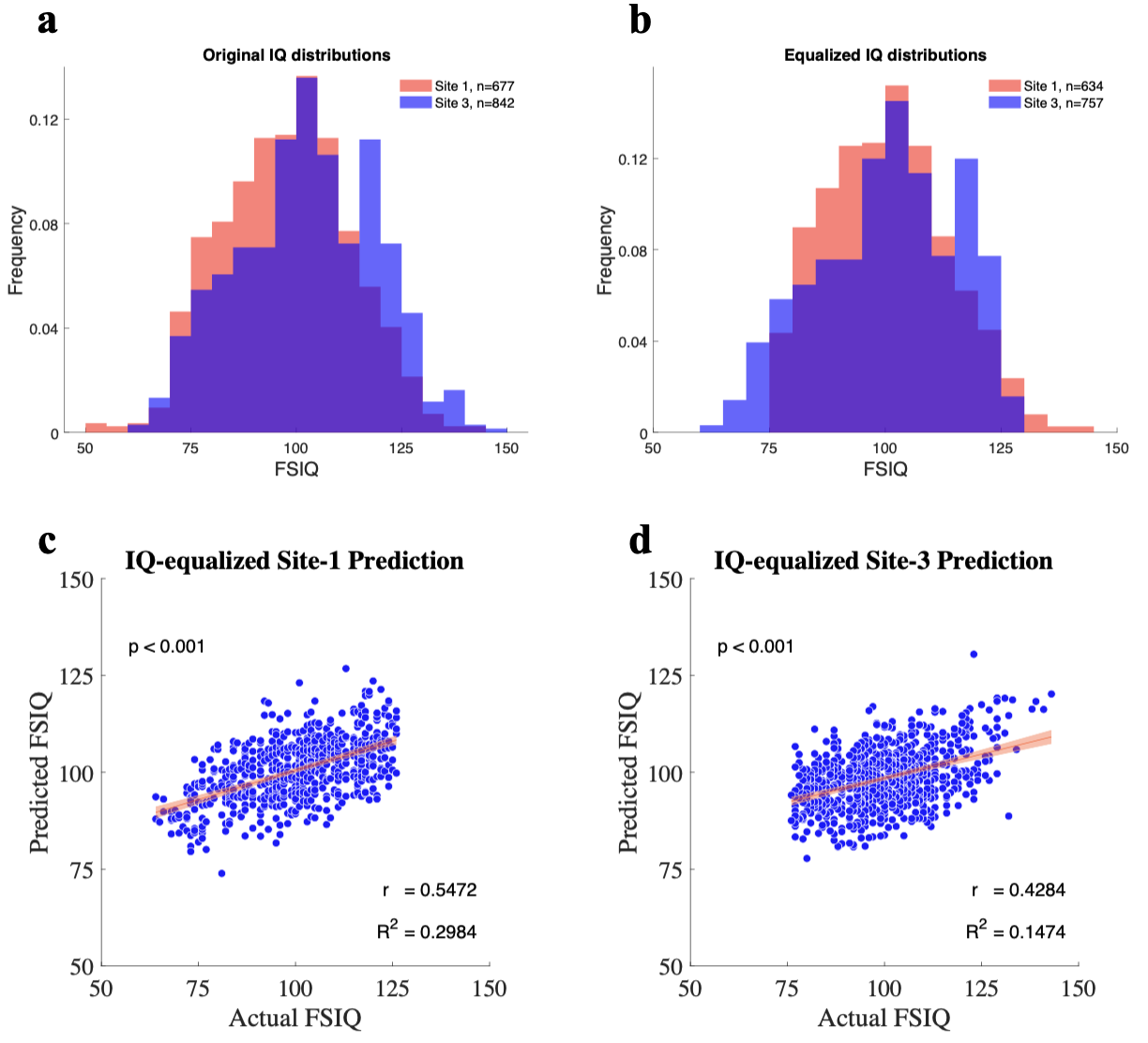
**

**Supplementary Figure 4 | Prediction performance difference across study sites.** We also investigated the difference we observed of FSIQ prediction performance across study sites. First, we examined the distribution of basic demographics (age and gender) at each site. We focused the analysis on site 1 and site 3 as they consist of most of the samples (92% in total) to simplify the statistics. No significant difference of age (Wilcoxon’s test: p=0.5362) and gender (Chi-squared=0.0245, p=0.8755) distribution was observed across study sites. We then conducted a correlation analysis to assess the effects of head motion on the preprocessed fMRI connectome, which showed no correlation between frame-wise displacement and FSIQ residuals (r=0.0379, p<0.05), thus confirming that the performance difference across study sites was not caused by issue of data quality. We also checked that the proportions of the most common psychiatric disorders were equivalent in study sites (Supplementary Table 1). **(a)** Subsequently, we examined the FSIQ distribution of each site and found their distributions have significant differences (site 1’s mean=102.05, site 3’s mean=97.14, Wilcoxon’s test p<0.001). **(b)** We equalized their FSIQ distributions by excluding subjects with highest IQs in site 1 and subjects with lowest IQs in site 3 such that resultant down-sampled populations both had a mean IQ of 100 (Wilcoxon’s test p=0.3266). **(c-d)** Finally, we evaluated and compared the predictability of the FSIQ prediction model on site 1 and site 3 with the equalized FSIQ distribution. As results, their corresponding leave-one-site-out models were significantly predictive to the distribution-modified unseen data and still performed differently with each other (Fisher’s z=3.02, P=0.0025), suggesting that the site effect we previously observed was not entirely, if partly, due to the difference in IQ distributions. Hence, we concluded that the site effect was not because of data quality nor any distribution difference of variables in our scope.


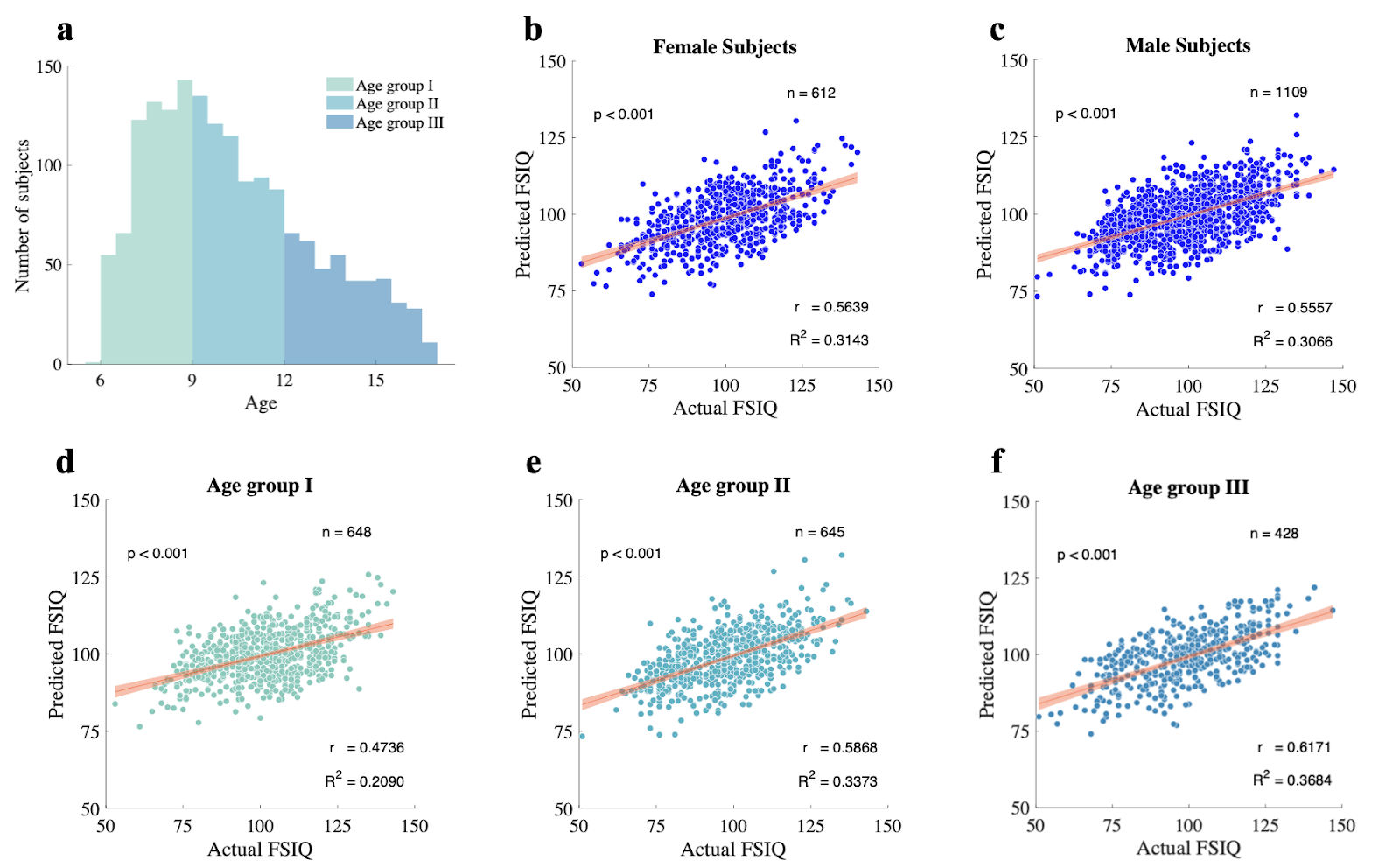


**Supplementary Figure 5 | FSIQ prediction across gender and age. (a)** Subjects were divided into three age groups (group I: age < 9, group II: age between 9 and 12, group III: age > 12) based on the brain development speed^77^ and to balance the sample size of each age group (n_1_=648, n_2_=645, n_3_=428). **(b-f)** The model predicted FSIQ with reasonably high performance for all gender and age groups. The predictability had no significant correlation with gender (Fisher’s z= 0.24, P=0.8103) whereas the FSIQ predictability was significantly higher for elder subjects than younger subjects (between group I and II: Fisher’s z=2.84, P=0.0045; between group I and III: Fisher’s z=3.29, P=0.001), though group II and III had similar predictability (Fisher’s z=0.76, P=0.4473).


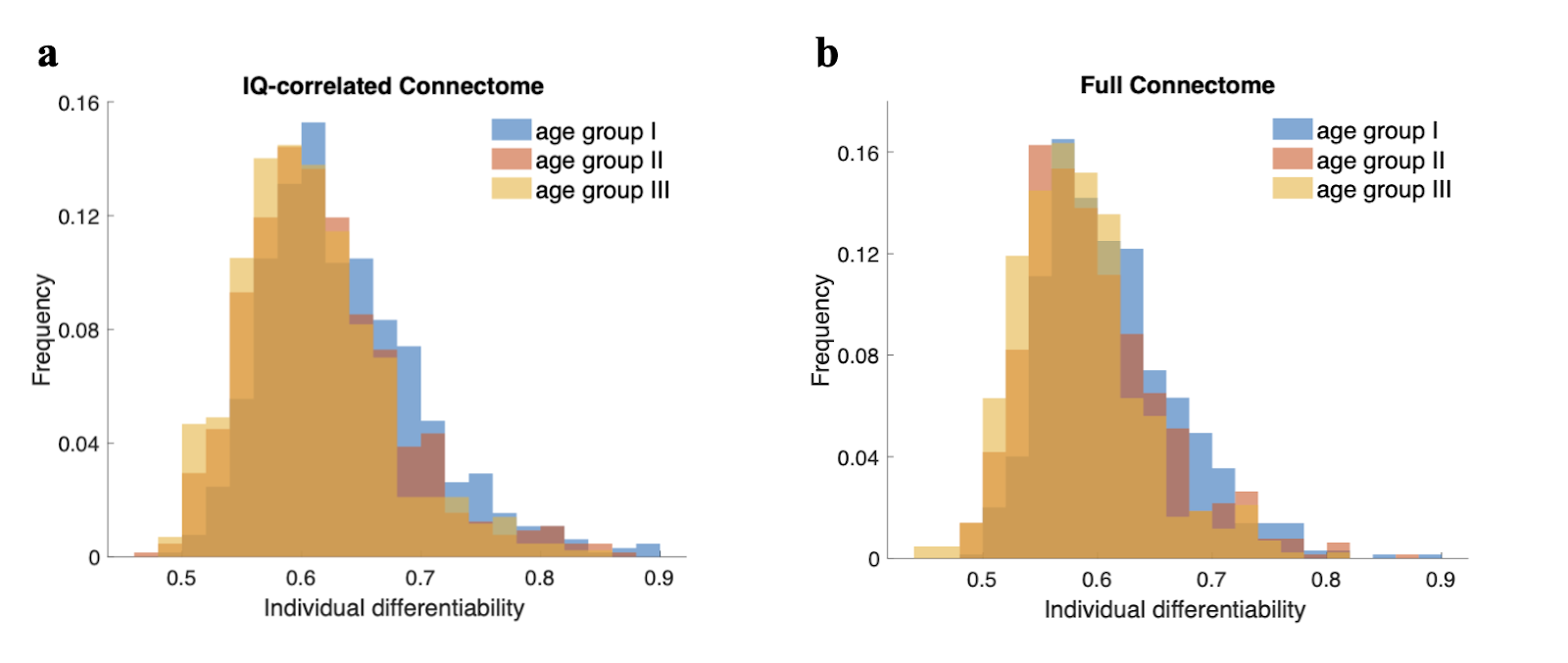


**Supplementary Figure 6 | Individual differentiability of functional connectome across age.** As age showed no correlation with FSIQ values (r=-0.0097, p<0.001), we hypothesized that the decrease of predictability in younger subjects was due to higher variance of brain connectivity in younger subjects compared with elder subjects. Individual differentiability was proposed to quantitatively assess the deviation of each subject from the population average (see Methods for details). **(a)** We obtained distributions of IQ-correlated individual differentiability for each age group (group I’s mean: 0.6364, group II’s mean=0.6189, group III’s mean=0.6080) and comparing individual differentiability distributions across age groups indicated significant differences between them (Wilcoxon’s test. group I and II: p<0.001; group I and III: p<0.001), which suggested that the variance of FSIQ-correlated connectome in elder subjects was indeed lower than younger subjects. **(b)** We further investigated whether the difference in connectome variance was IQ-specific or was general for the whole connectome. Another individual differentiability distribution was calculated using the full connectome (group I’s mean: 0.6128, group II’s mean=0.5976, group III’s mean=0.5844), and the significant difference in individual differentiability across ages retained (Wilcoxon’s test. group I and II: p<0.001; group I and III: p<0.001). Together, these results suggested that the positive correlation between FSIQ predictability and age was attributed to a decrease of individual differentiability in the whole connectome for elder subjects.


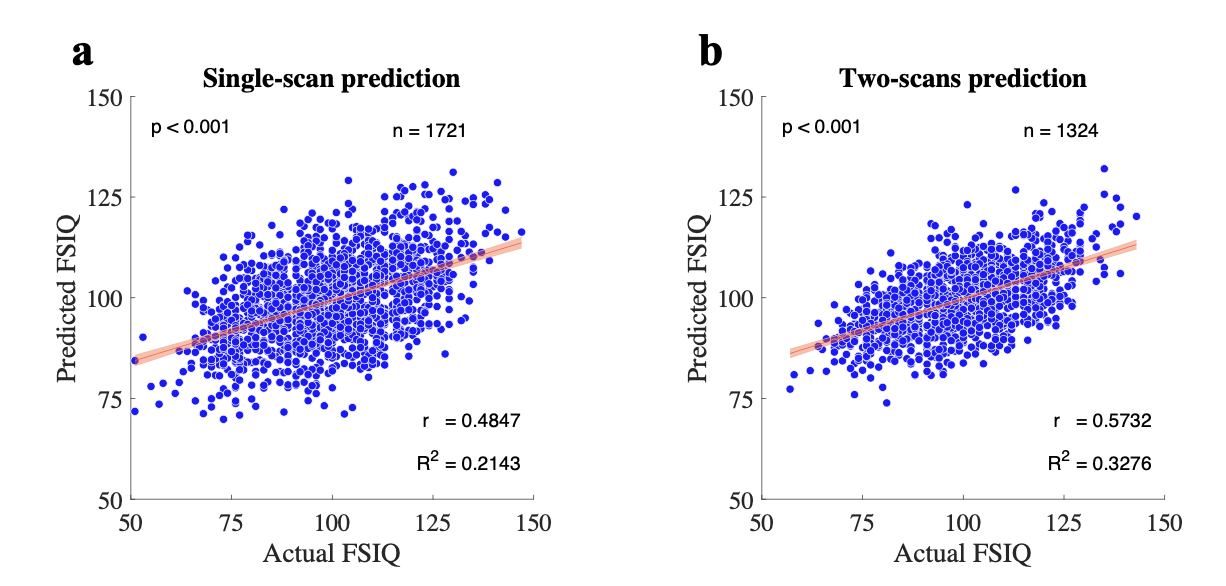


**Supplementary Figure 7 | Prediction reliability enhanced by additional runs of rsfMRI scans.** To assess the reliability of FSIQ prediction models, we tested the effects of additional fMRI runs on FSIQ prediction and model training. To examine the effects of additional rsfMRI runs on FSIQ prediction, we respectively evaluated the prediction performance on all subjects using only data from the first fMRI run and on subjects having two fMRI runs using both runs. Compared with predictions derived by only one fMRI run in **(a)**, predictions yielded using two fMRI runs in **(b)** showed significantly enhanced prediction power (Fisher’s z=3.37, P<0.001), suggesting that training models with additional fMRI runs help improve the reliability of FSIQ prediction.


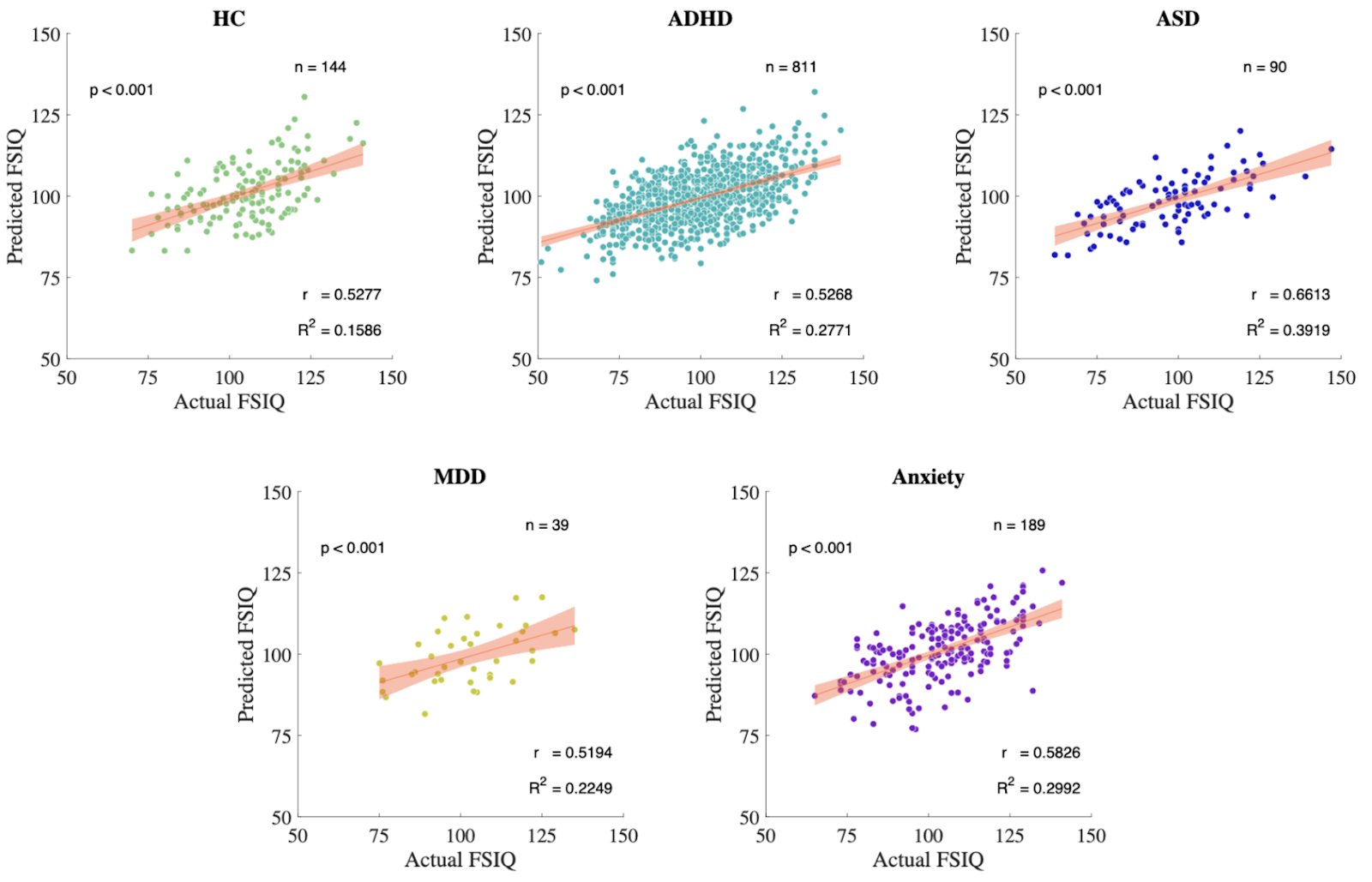


**Supplementary Figure 8 | FSIQ prediction for each diagnosis group.** The diagnosis-specific predictability is evaluated as the performance of the “standard model” on each of the diagnosis groups.


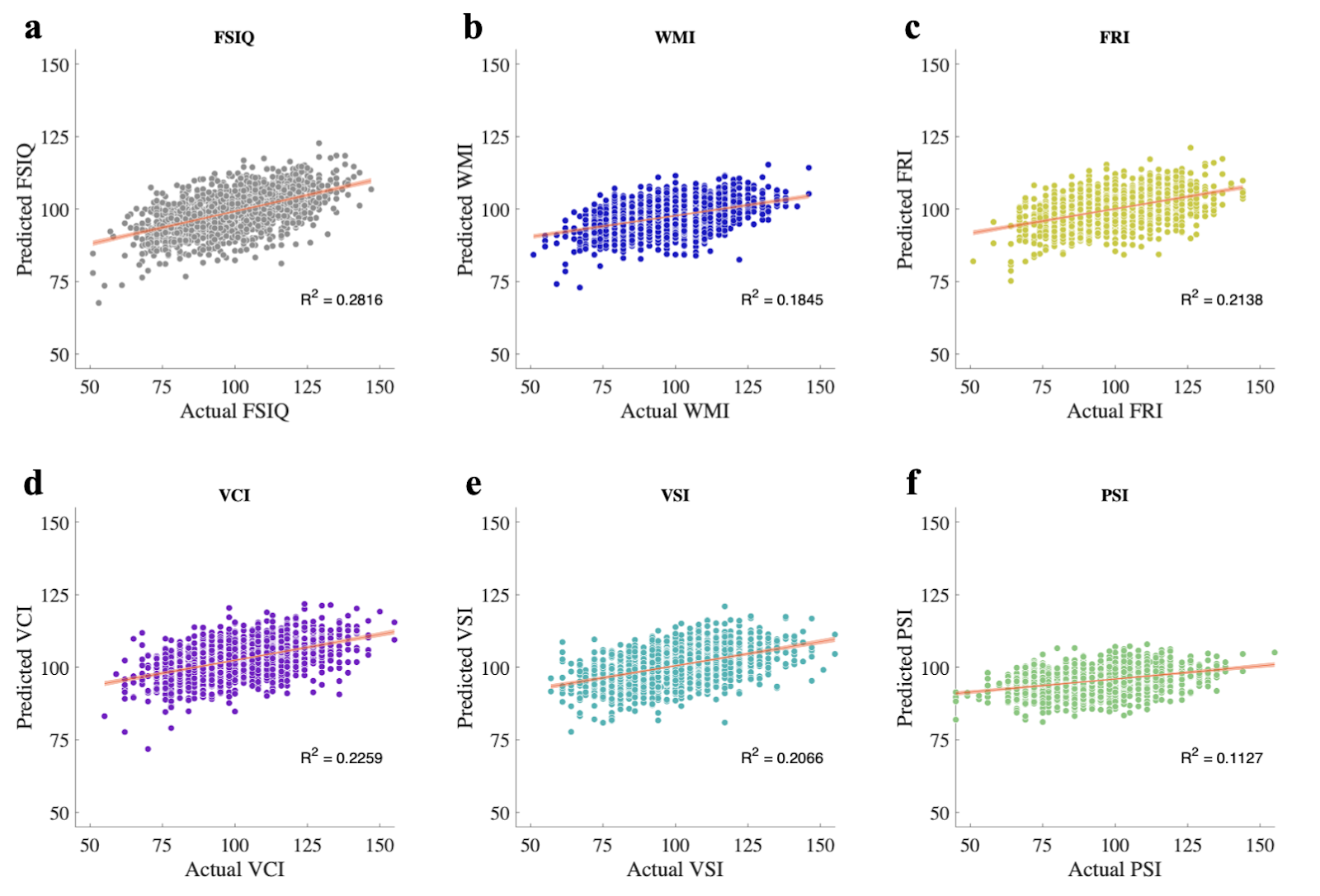


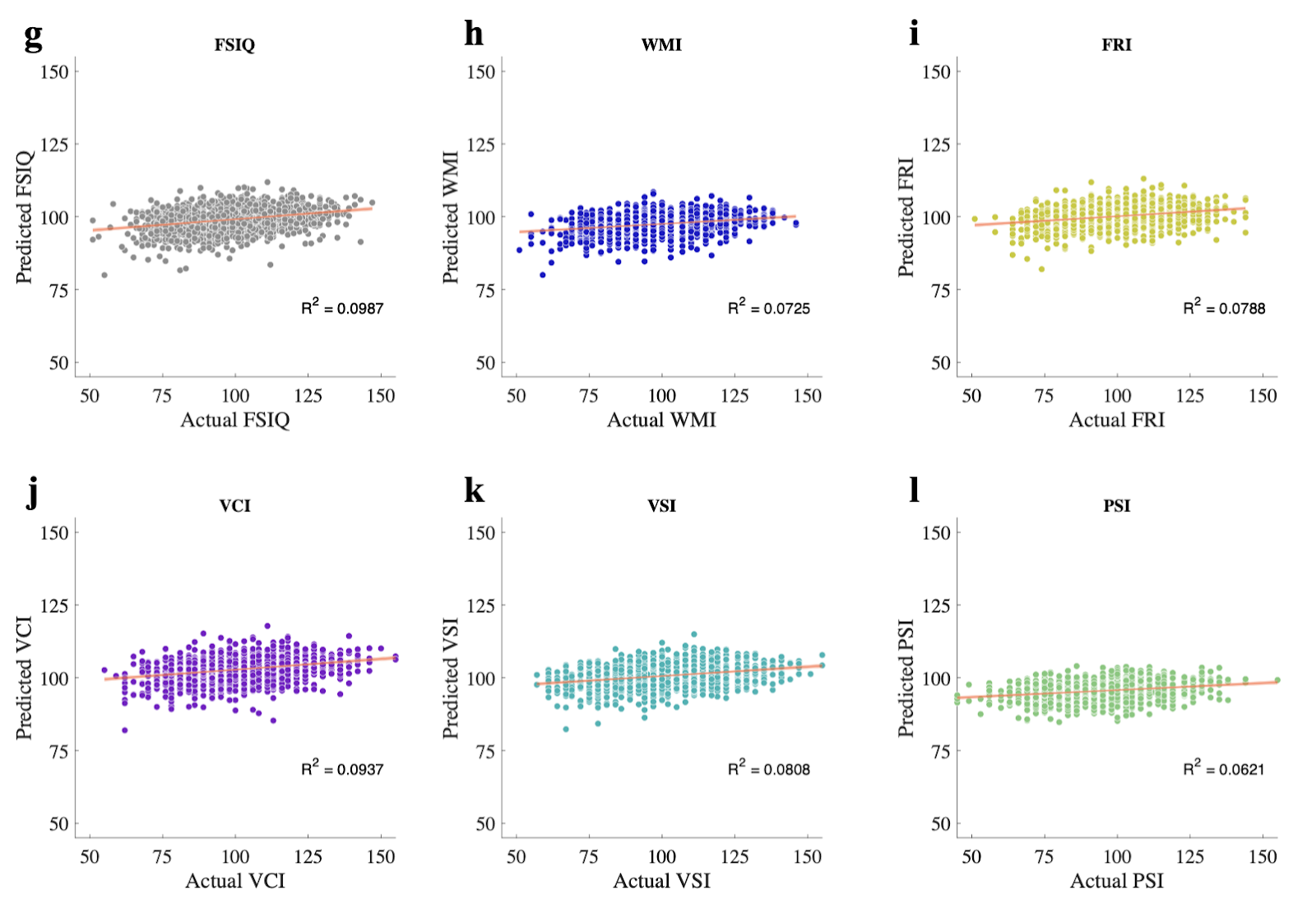


**Supplementary Figure 9 | Correlations between ROI importance and cognitive measures. (a-f)** Correlations between IQ-correlated ROI importance and cognitive measures. IQ-correlated ROI importance is defined as the average of feature weights of IQ-correlated connections involving an ROI. The feature weights are derived from the unified FSIQ prediction model.

**(g-l)** Correlations between full ROI importance and cognitive measures. Full ROI importance is defined as the average of feature weights of all connections involving an ROI. The correlations are calculated as the true-predicted correlation derived by multiple linear regression models.

**Supplementary Table 1** Diagnosis distribution across main study sites

|  | Site 1 (N=677) | Site 3 (N=842) | Stats |
| --- | --- | --- | --- |
| HC | n=51 | n=82 | Chi-squared=2.2848, p=0.1306 |
| ADHD | n=279 | n=316 | Chi-squared=2.1347, p=0.1440 |
| ASD | n=57 | n=72 | Chi-squared=0.0084, p=0.9272 |
| MDD | n=14 | n=22 | Chi-squared=0.4815, p=0.4877 |
| Anxiety | n=55 | n=74 | Chi-squared=0.2132, p=0.6442 |

Frequency of each diagnosis group of interest (HC, ADHD, ASD, MDD and anxiety) does not significantly differ between the two main study sites, indicating the transdiagnostic populations in these study sites are statistically homogeneous.

|  | HC | ADHD | ASD | MDD | Anxiety | All Subjects |
| --- | --- | --- | --- | --- | --- | --- |
| Visual | z=0.27, P=0.7872 | z=1.86, P=0.0629 | z=1.54, P=0.1236 | z=0.42, P=0.6742 | z=0.91, P=0.3628 | **z=2.73, P=0.0063** |
| Motor | z=0.89, P=0.3735 | z=0.93, P=0.3524 | z=1.22, P=0.2225 | z=0.19, P=0.8493 | z=0.32, P=0.7490 | z=1.75, P=0.0801 |
| DAN | z=0.54, P=0.5892 | **z=3.21, P=0.0013** | z=0.36, P=0.7188 | z=0.62, P=0.5353 | z=0.36, P=0.7188 | **z=4.23, P<0.0001** |
| VAN | z=0.72, P=0.4715 | z=1.11, P=0.2670 | z=0.67, P=0.5029 | z=-0.21, P=0.8337 | z=0.32, P=0.7490 | z=1.86, P=0.0629 |
| LIMBIC | z=0.27, P=0.7872 | z=0.11, P=0.9124 | z=0.11, P=0.9124 | z=-0.07, P=0.9442 | z=-0.18, P=0.8572 | z=0.37, P=0.7114 |
| FP | z=0.85, P=0.3953 | **z=2.30, P=0.0214** | z=1.12, P=0.2627 | z=-0.06, P=0.9522 | z=0.53, P=0.5961 | **z=3.73, P=0.0002** |
| DMN | z=1.41, P=0.1585 | **z=3.36, P=0.0008** | z=0.54, P=0.5892 | z=0.42, P=0.6745 | z=1.16, P=0.2460 | **z=4.78, P<0.0001** |

**Supplementary Table 2** Network importance with respect to FSIQ prediction across diagnosis

Interestingly, we discovered that brain networks possessed diagnosis-specific effects on FSIQ. For ADHD patients, DAN, FPCN and DMN were identified as significantly influential networks to FSIQ. For other diagnosis groups, as considerable changes of predictability were observed, more data was needed to address the significance issue as we were unable to find any significantly influential network because of their sample sizes. Nevertheless, we highlighted the networks with p-values lower than 0.5 (*Italics* in the table), which means they had more chance to have significant effects on the prediction model than no effect. We defined these *italic* insignificant networks as candidates of influential networks. Although we were unable to confirm their significance, these influential network candidates provided us with informative insights into the diagnosis-specific contribution of each brain network to intelligence. For instances, visual network, somatomotor network and FPCN were most influential networks to FSIQ for ASD patients, while visual network and DMN were the identified networks for patients of anxiety disorders. Network importance of each of the brain networks is derived by a set of Fisher’s z tests between the “standard model” and leave-brain-network-out models. **Bolded** inputs indicate significant results (p<0.05). P-values are uncorrected.

**Supplementary Table 3** Replication of FSIQ prediction on independent cohorts with data harmonization

| Cohort | ADHD-200 | | ABIDE I | | ABIDE II | |
| --- | --- | --- | --- | --- | --- | --- |
|  | r | P | r | P | r | P |
| No harmonization | 0.1983 | 8.20 x 10^-8^ | 0.1945 | 3.08 x 10^-10^ | 0.2344 | 1.35 x 10^-12^ |
| Harmonization - No covariate | 0.1990 | 7.41 x 10^-8^ | 0.1972 | 1.73 x 10^-10^ | 0.2333 | 1.73 x 10^-12^ |
| Harmonization - Age as covariate | 0.1992 | 7.23 x 10^-8^ | 0.1975 | 1.62 x 10^-10^ | 0.2333 | 1.70 x 10^-12^ |

Data harmonization procedure was implemented to investigate potential site effect of independent cohorts. The data harmonization was conducted with or without age as a covariate. We selected age as a potential covariate because age was shown to influence FSIQ predictability (Supplementary Figure 5), thus harmonizing age out of the data might affect model performance. Nevertheless, harmonized data (with age as a covariate or not) showed very similar FSIQ predictability with unharmonized data under the HBN-trained prediction model, partly explained by the fact that the age and FSIQ distributions were comparable across all four independent cohorts (Table 1). Hence, to simplify the procedure and accommodate as many subjects as possible, we propose to not include a data harmonization step in this framework. That being said, we recognize the potential benefits of data harmonization to mitigate inter-cohort bias. Additionally, we do suggest the implementation of data harmonization when the data distributions showed significant discrepancy and acknowledge the possibility that a more advanced procedure, such as transfer learning, may further address the site effects.
